# Supplementary material for: Mouse REC114 is essential for meiotic DNA double-strand break formation and forms a complex with MEI4
Source: Life Sci Alliance. 2018 Dec 10;1(6):e201800259. doi: 10.26508/lsa.201800259 (PMC6288613; doi:10.26508/lsa.201800259)
Supplement: Supplementary file 1 [file LSA-2018-00259_TableS1.docx]

Supplementary Table 1. List of oligonucleotides used for RT-PCR.

| Oligonucleotide |  |
| --- | --- |
| 16U22 | ATG TCT GAA GCG GGA AAT GTG G |
| 16U21 | ATG TCT GAA GCG GGA AAT GTG |
| 232L22 | TCT GCC CTT GGG AAA TGA AGA A |
| 644L22 | TAG GCG AGG GTC AGC TTC TCT G |
| 688L24 | GCG TAG AAA AGG TCC CAA TTC TTC |
| 695U22 | TGG GAC CTT TTC TAC GCT TAT G |
| 806L25 | CCT TCA GTT CCT TAG TTG CAT ACA T |
